# Reading the Fog: Real-Time LiDAR-Based Detection Range Estimation for Autonomous Vehicles in Adverse Weather

**DOI:** 10.3390/s26206623

**Published:** 2026-10-19

**Authors:** Jongmin Lee

**Affiliations:** 1 Department of Mechanical Engineering, Seoul National University, Seoul 08826, Republic of Korea; jesus911021@snu.ac.kr; 2 Autonomous Driving SW Division, Hyundai Mobis Company Ltd., Seoul 16891, Republic of Korea

**Keywords:** LiDAR, autonomous driving, adverse weather, fog, detection range, visibility estimation, real-time perception, fail-operational

## Abstract

**Highlights:**

**What are the main findings?**

- LiDAR fog returns, conventionally treated as noise to be denoised, can be read directly as an optical measurement of how far the sensor can still see, yielding a per-frame maximum detection range, MDR^ (in meters), without any additional inference cost on top of an existing fog-denoising network.
- On two independent real-vehicle heavy-fog recordings, the recovered fog density β^_ext_ agrees to 0.21–0.22 m^−1^ across the recordings (MDR^ ≈ 17–19 m), and on the scenario corpus, a leave-one-out per-frame regression recovers the first-detection distance to within 1.99 m of the mean absolute error (R^2^ = 0.741, a 58% MAE improvement over the no-feature baseline) at the LiDAR scan rate (≈14 Hz).

**What are the implications of the main findings?**

- The proposed maximum detection range scalar provides exactly the per-frame, traceable, bounded visibility input that ISO 26262 hazard analysis, ISO 21448 SOTIF Clause 7 triggering-condition specification, and SAE J3016 Operational Design Domain definitions all require, making the perception-layer visibility signal directly consumable by safety arguments rather than only by downstream object detectors.
- Because the visibility estimator reuses the segmentation output that any fog-denoising stack already produces, the proposed pipeline is a drop-in upgrade for existing LiDAR perception systems: the cleaned cloud continues to feed the object detector while the previously discarded fog-labeled subset feeds a new MDR^ head, enabling safety controllers (e.g., speed capping and emergency pull-over) to react to silent detector failures before they propagate to vehicle behavior.

**Abstract:**

Detecting objects with LiDAR in fog has long been treated as a noise-removal problem: identify the fog returns and throw them away. This work takes the opposite view. The same fog returns that other methods discard are read as a direct measurement of how far the sensor can still see. After an off-the-shelf fog-segmentation step, the near-range fog points are used to estimate the atmosphere’s optical thickness, which is then converted into a per-frame maximum detection range (MDR)—a single, human-readable number, in meters, that tells planning and safety modules how far the LiDAR is currently usable. The estimator has no learnable parameter (the physics core—a per-frame Beer–Lambert slope fit, followed by the Koschmieder mapping) and runs in real time during driving. We validate it with real-vehicle experiments in controlled heavy-fog scenarios. Across six fog runs, the estimated MDR recovers the true first-detection distance with a mean absolute error of 1.99 m and R^2^ = 0.741—a 58% error reduction over a no-feature baseline. To rule out the possibility that the result is an artifact of our own recording, the same estimate is cross-checked against two independent references used by the wider community: the STF transmissometer dataset and the Hahner et al. automotive fog simulator. Our estimate agrees with both, which is its key differentiator from prior LiDAR-fog work that reports performance only on its own data. The result is a real-time, parameter-free, externally corroborated visibility readout that fits inside an existing fog-denoising pipeline and gives downstream autonomy a quantitative answer to the question: “How far can I see right now?”

## 1. Introduction

Adverse weather is one of the longest-standing open problems of LiDAR-based autonomous driving. The dominant body of work treats fog, snow, and rain as *noise*—physical returns that contaminate the point cloud and must be removed before downstream object detection. Per-point denoising classifiers have steadily improved in this framing, yet the underlying premise—fog is a contaminant—has remained unchallenged. The present work proposes different premises. Once fog returns have been segmented from real returns by the same per-point classifier the denoiser would use, the very same segmented set carries a different kind of information: how far the sensor can still see through the current fog. The denoiser removes fog, and the proposed pipeline reads it.

The importance of this distinction extends beyond producing a cleaner point cloud. For safe autonomous driving, it is essential to understand the limitations of sensor perception under adverse weather conditions. The maximum detection range (MDR)—defined here as the farthest range at which the LiDAR can still register a real object return given the current weather state—provides a quantitative measure of the sensor’s effective perception range. Such information can support the assessment of perception performance and operational limitations under varying environmental conditions. Rather than relying solely on a denoised point cloud, the proposed approach provides a quantitative, per-frame estimate of detection range in meters. Producing this information directly from the LiDAR point cloud, at the sensor frame rate, is the goal of this work.

Concretely, the per-point intensity I(r) of the fog-labeled subset within the ego-proximal volume r ∈ [0.5, 3] m—a region that, on the dual-LiDAR roof-mount instrumented vehicle used in this work, contains a dense and contamination-free fog return—is collected, and log I(r) is fit linearly against r. By the Beer–Lambert law, the negative half-slope of this fit is the extinction coefficient β^_ext_, and the Koschmieder relation yields the maximum detection range as MDR^ = 3.912/β^_ext_ directly, with no concentric-ring partition, no ring-radius tuning, and no engineered density vector. A ±5-frame median over per-frame slopes suppresses single-frame jitter without introducing any temporal model. A consequence of this design is that the fog-segmentation network—the same network the denoising community already runs at the LiDAR front-end—becomes *dual-purpose*: a single forward pass yields both the cleaned cloud (the conventional product) and the per-frame visibility signal (the product of this work) at no additional inference cost.

Two further design choices distinguish the present work. First, the entire chain—the raw dual roof-mounted LiDAR point cloud, fog segmentation, ego-proximal window selection, Beer–Lambert slope fit, and Koschmieder mapping—runs in real time on a single in-vehicle desktop-class GPU (NVIDIA RTX 2070, 8 GB VRAM). On-vehicle measured end-to-end latency is 60–70 ms per frame, comfortably within the LiDAR scan cycle (≈70 ms, ≈14 Hz). Second, the estimator is validated on real-vehicle heavy-fog data acquired on a closed instrumented track (FMTC, SNU) by the SNU instrumented test vehicle, with two independent reference recordings yielding cross-recording-concordant β^_ext_ (Section 4). The dataset is released on Hugging Face [1,2] for community benchmarking—an asset that the adverse-weather LiDAR community has long lacked outside of simulated or geographically narrow public sources.

Contributions of this work. This paper makes the following contributions, organized so that each item points to the section in which it is developed.

(i) **Reframing of LiDAR fog perception (**Section 3**).** This paper recasts adverse-weather LiDAR perception from *fog removal* to *fog reading*. The fog-labeled returns produced by an existing denoising network—conventionally discarded as noise—are read here as the visibility signal itself, which is the central methodological inversion of this work.
(ii) **Direct extinction estimator without ring engineering (**Section 3.2**).** The fog density, expressed as the Beer–Lambert extinction coefficient β^_ext_ (in m^−1^, with larger values meaning denser fog), is recovered as the negative half-slope of log I(r) on the fog-labeled subset within an ego-proximal window r ∈ [0.5, 3] m. The estimator uses no concentric-ring partition, no per-ring radius schedule, and no engineered density vector; the window bounds are dictated by the geometry of the dual roof-mount, not tuned. A ±5-frame temporal median suppresses single-frame jitter without introducing any sequence model.
(iii) **The dual-purpose use of the segmentation network (**Section 3.1**).** The same fog-segmentation network that any denoising stack already runs is used here for two purposes simultaneously: its output feeds (i) the conventional cleaned cloud consumed by downstream object detectors and (ii) the fog-labeled cloud consumed by the visibility estimator. The visibility estimator, therefore, adds zero inference cost on top of an existing denoising pipeline—a drop-in upgrade.
(iv) **Koschmieder-grounded mapping to a maximum detection range scalar (**Section 3.3**).** The extinction coefficient β^_ext_ is mapped to a per-frame maximum detection range, MDR^ = 3.912/β^_ext_ (in meters), via the Koschmieder relation under the standard 5% contrast threshold. The mapping introduces no further tunable parameter, and the only constant is the textbook Koschmieder coefficient 3.912 = ln(1/0.05).
(v) **Standards-aligned auditable output (**Section 3.3 and Section 5**).** The MDR^ is precisely the per-frame, traceable, reproducible, bounded scalar that ISO 26262 hazard analysis (exposure-class assignment), ISO 21448 SOTIF Clause 7 (triggering-condition specification), and SAE J3016 ODD definition (visibility envelope) all require. Every step of the chain—segmentation output, linear fit, constant divide, and median—is inspectable, which a black-box deep regressor would not satisfy.
(vi) **Cross-recording and cross-instrument concordance (**Section 4**).** On two independent real-vehicle heavy-fog reference recordings, the per-frame median β^_ext_ agrees to 0.21–0.22 m^−1^ across recordings (MDR^ ≈ 17–19 m), supporting a sensor-universal fog signature rather than a recording-specific artifact. The recovered β^_ext_ is additionally cross-validated against (a) the SeeingThroughFog on-vehicle transmissometer [3] for cross-sensor/cross-site agreement and (b) the Hahner et al. [4] automotive fog forward-simulator [4] for fog-regime placement, evidencing that the measurement tracks the underlying optical state of the atmosphere, as quantified by independent instruments and established physical models.
(vii) **Real-time on-vehicle deployment (**Section 3.5 and Section 4**).** The full A1–A5 chain runs end-to-end on an in-vehicle desktop-class GPU (NVIDIA RTX 2070, 8 GB VRAM) at 60–70 ms per frame within the LiDAR scan cycle.
(viii) **Public real-vehicle heavy-fog dataset release (**Section 4**).** The real-vehicle heavy-fog dataset used in this work is released on Hugging Face [1,2] for community benchmarking.

**Scope.** This paper addresses fog. The term “adverse weather” in the title denotes the motivating safety context, in line with much of the prior LiDAR-fog literature, but all experiments, modeling, and validation in this paper are restricted to fog conditions. Extension to rain and snow—where Mie- versus Rayleigh-regime scattering produces qualitatively different LiDAR returns.

## 2. Related Work

Recent research on LiDAR perception under adverse weather has been dominated by efforts to *remove* fog from the point cloud rather than to *read* fog from the point cloud as a signal in its own right. Dreissig et al. surveyed the field and organized prior work along three axes—adverse-weather data, point-cloud manipulation and denoising, and robust perception with sensor fusion—and explicitly identified the quantification of weather-induced perception degradation as an open problem [5]. Their review makes clear that fog, rain, and snow do not merely add noise: they alter point density, intensity, and spatial structure through scattering and occlusion in ways that are range-dependent and, therefore, policy-relevant for an autonomous vehicle [5].

Within the denoising line, several recent studies have treated adverse-weather restoration as a preprocessing stage for downstream perception. Zhao et al. proposed TripleMixer, a plug-and-play 3D point–cloud denoiser that integrates spatial, frequency, and channel mixers, and released the simulated Weather-KITTI and Weather-NuScenes benchmarks, together with denoising, semantic-segmentation, place-recognition, and detection evaluations [6]. Teufel et al. extended this idea into the cooperative–perception setting with DenoiseCP-Net, which folds voxel-level noise filtering and 3D object detection into a single sparse-convolution backbone, reducing inter-vehicle bandwidth by up to 23.6% on an OPV2V extension [7]. Park and Shim further removed the supervision burden with LIORNet, a U-Net++ snow-removal network trained from physically motivated pseudo-labels (range-dependent intensity, snow reflectivity, sparsity, and sensing-range constraints) on WADS and CADC [8]. Earlier in the same thread, Piroli et al. reframed weather-effect detection as energy-based outlier detection, released the SemanticSpray dataset [9], and subsequently showed that few-shot plus semi-supervised pseudo-labeling can reach near-supervised accuracy on snow/fog/spray with a fraction of labels [10]. Beyond per-point denoising, Yang et al.’s GRC decouples geometric and reflectance branches and reports state-of-the-art adverse-weather segmentation without any weather simulation or augmentation [11]; Huang et al.’s L4DR (AAAI 2025 Oral) fuses LiDAR with a 4D radar and recovers a +20% 3D mAP under simulated fog [12]; and Xiao et al.’s SemanticSTF (CVPR 2023) provides the first densely point-wise-annotated real adverse-weather dataset for both domain-adaptive and domain-generalizable 3D semantic segmentation [13]. Across these works, the common goal is to keep degraded perception working; **none of them produce a vehicle-level scalar that tells the planner how far the LiDAR can still be trusted on the next frame.**

A second relevant line shifts attention from object-centric outputs to dense volumetric scene representations. Wei et al.’s SurroundOcc (ICCV 2023) lifts multi-camera features to a 3D volume with 2D-3D attention and constructs dense occupancy ground truth by Poisson-reconstructing fused multi-frame LiDAR scans [14]. Zhang et al.’s OccFormer proposes a dual-path local/global transformer over the camera-derived 3D voxel grid with a Mask2Former-style decoder that mitigates voxel sparsity and class imbalance [15]. Tian et al.’s Occ3D [16] standardizes this task by releasing visibility-aware occupancy labels on Waymo Open and nuScenes, together with a coarse-to-fine baseline (CTF-Occ) [16]. These dense representations are conceptually adjacent to our work—both summarize the surrounding scene on a regular grid—but they are designed to describe geometry and semantics under nominal conditions, not to quantify how that grid degrades in fog [14,15,16].

A third, smaller line directly stress-tests perception stacks against degradation. Hao et al.’s MSC-Bench introduces 16 corruption combinations spanning camera and LiDAR inputs (including adverse-weather corruptions) and reports substantial mAP drops across six 3D detectors and four HD-map models, framing multi-sensor degradation as an explicit safety concern [17]. Together with the survey by Dreissig et al. [5], this line of work establishes that stacks *degrade* under adverse weather, but evaluates model accuracy, not a per-frame scalar that the planner can act on.

Taken together, the post-2023 literature has substantially advanced adverse-weather denoising/restoration, dense-occupancy reasoning, and perception-stack robustness benchmarking, but a gap remains between these perception outputs and a directly usable safety-facing range quantity. Existing methods aim to recover cleaner point clouds [6,7,8,9,10,11,12,13], richer 3D scene representations [14,15,16], or more robust diagnostics [5,17] for downstream tasks, such as detection, segmentation, and recognition. Standards reinforce the same gap: ISO 21448 (SOTIF) and SAE J3016 require an Operational Design Domain that bounds where the system is intended to operate, but the ODD is normally specified as a nominal limit (e.g., visibility class), not as a per-frame, run-time scalar that the vehicle itself can compute. **In contrast, our work focuses on deriving a per-frame maximum detection range (MDR) signal from fog-related LiDAR evidence itself.** Rather than treating fog only as corruption to be removed, the proposed framework preserves fog-labeled returns within an ego-proximal window (radial distance r ∈ [0.5, 3] m, where the dual-roof-mount geometry yields a dense, contamination-free fog return), recovers the Beer–Lambert extinction coefficient β^_ext as the negative half-slope of log I(r), and maps it to MDR^ = 3.912/β^_ext via the standard Koschmieder relation, yielding a per-frame scalar MDR ∈ [0, 60] m at ≈14 Hz on an in-vehicle desktop-class GPU (NVIDIA RTX 2070, 8 GB VRAM), with no concentric-ring partition, no ring-radius hyperparameter, and no engineered density vector. This shifts the focus from weather-robust perception alone to perception outputs that can be directly consumed as an operational constraint in fog-aware autonomous driving—the missing observable that ISO 26262 functional-safety chains, ISO 21448 SOTIF perception monitors, and SAE J3016 ODD compliance checks can read at runtime. Recent benchmarking of 3D object detection under common corruptions in autonomous driving [18] has further documented that fog, rain, and snow induce qualitatively distinct degradation patterns, supporting our decision to treat fog as a self-contained problem in this work.

## 3. Method

The estimator core itself comprises a closed-form physics module (Beer–Lambert slope fit and Koschmieder mapping) with no learned parameters, with only an optional lightweight four-feature linear regressor fitted on top, using per-recording leave-one-out cross-validation. This section describes how the proposed pipeline converts a raw LiDAR point cloud into a per-frame estimate of the maximum detection range, MDR^, expressed in meters. The seven processing stages in the chain are walked through end-to-end (Section 3.1), and the two physics-based steps that perform the actual estimation are detailed in turn: the per-frame extraction of the fog extinction coefficient β^_ext_ (Section 3.2) and its conversion to MDR^ via the Koschmieder relation (Section 3.3). The remaining subsections describe the automatic ground-truth labeling protocol (Section 3.4) and the on-vehicle real-time deployment (Section 3.5). We emphasize that Stages A4–A5 in the pipeline introduce no learned parameters: the off-the-shelf segmentation network at Stage A3 is interchangeable and is not a contribution of this work, and the only fitted component in the entire framework is the four-feature linear regressor described later in this section.

### 3.1. Pipeline Overview

**Figure 1:**
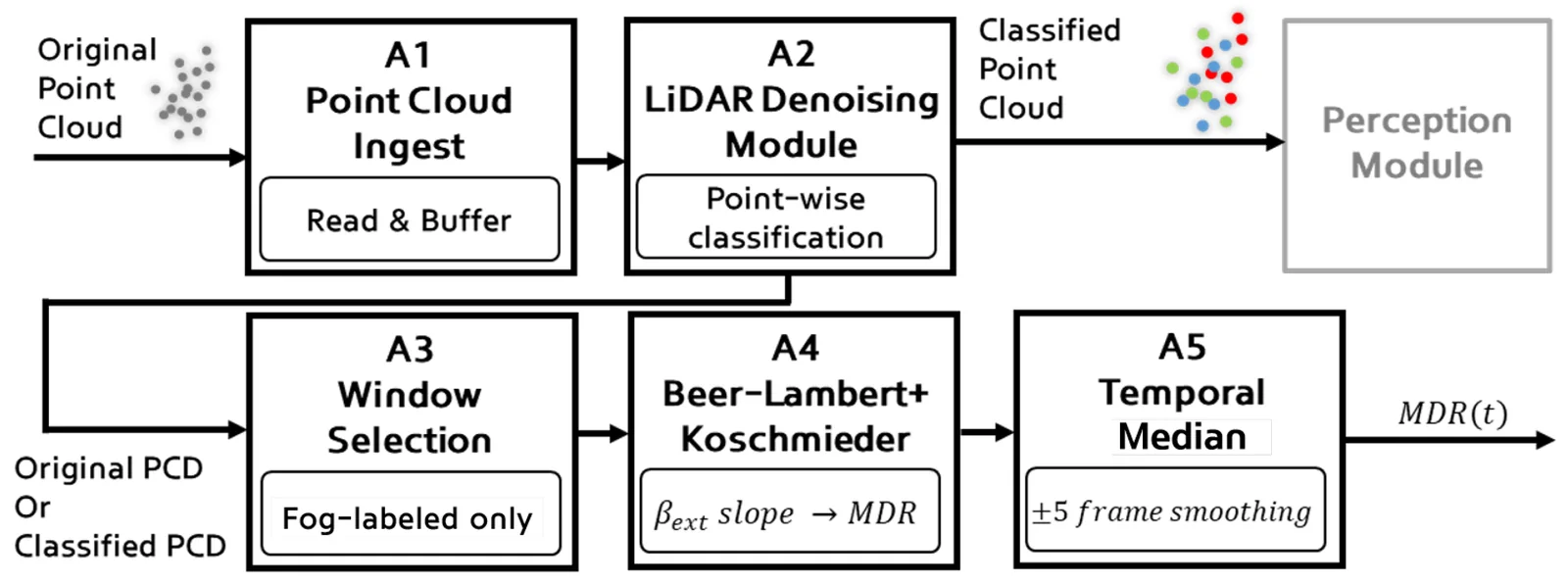
End-to-end pipeline overview. Stages A1–A5 from the raw dual roof-mounted LiDAR point cloud to the per-frame maximum detection range (MDR), executed on an in-vehicle desktop-class GPU at the LiDAR scan rate. The fog-segmentation network at A2 is dual-purpose (non-fog cloud → object detector; fog-labeled cloud → A3 ego-proximal window). Preprocessing (within-scan ego-motion compensation and ground removal) precedes A1 and is omitted from the diagram.

The full chain (Figure 1) consists of five stages, A1–A5, executed as a single forward pass per frame. Standard preprocessing steps (within-scan ego-motion compensation and ground removal) precede A1, but are not the contribution of this work and are omitted from the diagram. **A1. Point-cloud inges.** The raw point cloud is read from the dual roof-mounted Velodyne VLP-32C LiDARs, scanning at approximately 14 Hz. **A2. Fog/non-fog segmentation.** A pretrained network from prior work [19] labels every point as either *fog return* or *non-fog return*. Unlike the conventional adverse-weather denoising pipeline, which discards the fog-labeled subset as noise, the present pipeline retains it and uses it as the visibility signal—this distinction is the central methodological choice in this work. **A3. Window selection.** Only fog-labeled points within the radial range r ∈ [0.5, 3] m are retained; the rationale for this window is given in Section 3.2. **A4. Beer–Lambert fit and Koschmieder mapping.** A least-squares straight-line fit on log I(r) recovers the per-frame extinction coefficient β^_ext_ (Section 3.2), which is then converted to a maximum detection range, MDR^, via the Koschmieder relation (Section 3.3). **A5. Temporal median smoothing.** β^_ext_ is replaced by its median over a centered ±5-frame window before Koschmieder mapping is applied, yielding the per-frame MDR^(t). The chain contains no recurrence, no learned latent state, and no off-board call. The fog-segmentation network at stage A2 is dual-purpose: a single forward pass simultaneously produces (i) the conventional non-fog cloud consumed by downstream object detectors and (ii) the fog-labeled cloud consumed by the visibility estimator presented here. The proposed estimator, therefore, adds zero inference cost on top of any LiDAR stack that already performs fog denoising at A2.

### 3.2. Beer–Lambert Direct Extinction Estimation

The proposed pipeline summarizes the per-frame fog state by a single scalar, the extinction coefficient β_ext_ (units: m^−1^), which is large for dense fog and small for thin fog. β_ext_ is recovered directly from the fog-labeled points produced at stage A2, without any auxiliary feature engineering. Under the standard monostatic-backscatter form of the Beer–Lambert law, the LiDAR return intensity I from a fog volume at radial range r decays exponentially with r,

I(r) = I_0_ · β_back_ · exp(−2 β_ext_ · r) (1)

Equation (1) is the standard single-scatter form of the lidar equation for an attenuating medium [20]; it is the same Beer–Lambert attenuation that has been used to model lidar returns in fog, rain, and snow in prior adverse-weather lidar work [3,4]. Factor 2 in the exponent simply reflects round-trip travel: the pulse passes through the same fog volume twice (out and back). A larger β_ext_ means denser fog, which means the signal decays faster with range. Where I_0_ is the emitted intensity and β_back_ is the backscatter coefficient, taking the natural logarithm of both sides linearizes Equation (1) in r:

log I(r) = c − 2 β_ext_ · r (2)

Taking the log turns the exponential decay into a straight line, and the slope of that line is the only thing we need, as it gives β_ext_ directly. With c = log[I_0_ · β_back_] absorbed into the intercept, we fit Equation (2) by ordinary least squares over the fog-labeled points within the ego-proximal window r ∈ [0.5, 3] m and recover the per-frame extinction coefficient as β^_ext_ = −slope/2. The window itself is fixed by sensor geometry, not tuned: the lower bound (0.5 m) excludes returns from the vehicle body, and the upper bound (3 m) is where, on both reference recordings, the contamination-free dense-fog return ends and arbitrary scene objects begin to dominate the slope. Beyond 3 m, the monostatic-fog assumption underlying Equation (1) breaks down. The estimator, therefore, uses only what stage A2 already produces—the radial range and intensity of the fog-labeled subset—with no extra feature engineering and no per-ring radius schedule.

### 3.3. Koschmieder Mapping and Temporal Median

The extinction coefficient β^_ext_ obtained from Section 3.2 is converted to a maximum detection range via the Koschmieder relation. Under the standard meteorological-visibility convention (5% contrast threshold), the maximum detection range follows directly as

MDR^(t) = 3.912/β^_ext_(t) (3)

The constant 3.912 = ln(1/0.05) is fixed by the standard 5% meteorological contrast threshold and is not a tunable parameter. Equation (3) is the canonical Koschmieder visibility law [21,22], whose applicability to fog is extensively analyzed [23]. Intuitively, thinner fog means the signal travels farther before it is attenuated past the contrast threshold, so the detection range goes up roughly to 1/β_ext_—doubling the fog density roughly halves the sight distance. To suppress per-frame jitter from droplet-scale stochasticity, the pipeline applies a short-window temporal median to β^_ext_(t) before Equation (3); the window size and stage timings are reported in Table A1b. The median is a regularization step, not a sequence model—no recurrence, no learned state. Every step in the chain (segmentation → linear fit → constant divide → median) is inspectable on the bench, which is exactly what ISO 26262/ISO 21448 (SOTIF)/SAE J3016 require of a visibility input to the autonomous-driving stack.

### 3.4. Automatic Ground-Truth Labeling via Approach Scenarios

Per-frame ground-truth MDR (denoted MDR_GT_) is generated automatically from a controlled *approach scenario*, without manual bird’s-eye-view annotation. In each approach run, the ego vehicle—instrumented with the dual roof-mounted LiDAR—drives toward a stationary leading test vehicle along a closed-environment instrumented track under heavy fog, with the relative range monotonically decreasing from greater than 50 m to 0 m. An offline pretrained PointPillars detector [19], identical to the baseline used in our prior LiDAR adverse-weather work, is run frame-by-frame on the raw fog-degraded point cloud. The first frame in which the leading vehicle is successfully detected (above the operational confidence threshold) defines the per-episode MDR_GT_, taken to be the sensor-to-target ground-truth range at that frame. The range is recovered from the synchronized GNSS and vehicle-state logs of both vehicles, not from the LiDAR detection itself. Each approach traversal therefore yields one labeled *episode*—a single (MDR_GT_, β^_ext_) pair—and the labeled set is continuously expanded with each additional run. The contribution of this protocol is its labeling property, not its corpus size: the protocol is automatic (no human annotator in the loop), reproducible (deterministic, given the same approach run), and traceable (every MDR_GT_ links back to a logged GNSS range). These three properties—automation, reproducibility, and traceability—are precisely the ones required for the standards-aligned auditability claim of this work, and they are properties of the labeling protocol itself, rather than properties of any particular instance of it. Throughout this manuscript, we use three related terms: MDR denotes the conceptual maximum detection range; MDR^ denotes the per-frame estimate produced by our pipeline; and MDR_GT denotes the empirical ground-truth value defined above, obtained from the first frame in which the leading-vehicle detector reaches its operational confidence threshold of 0.5 (the default threshold of the upstream detector used in our acquisition stack).

### 3.5. On-Vehicle Real-Time Execution

**Figure 2:**
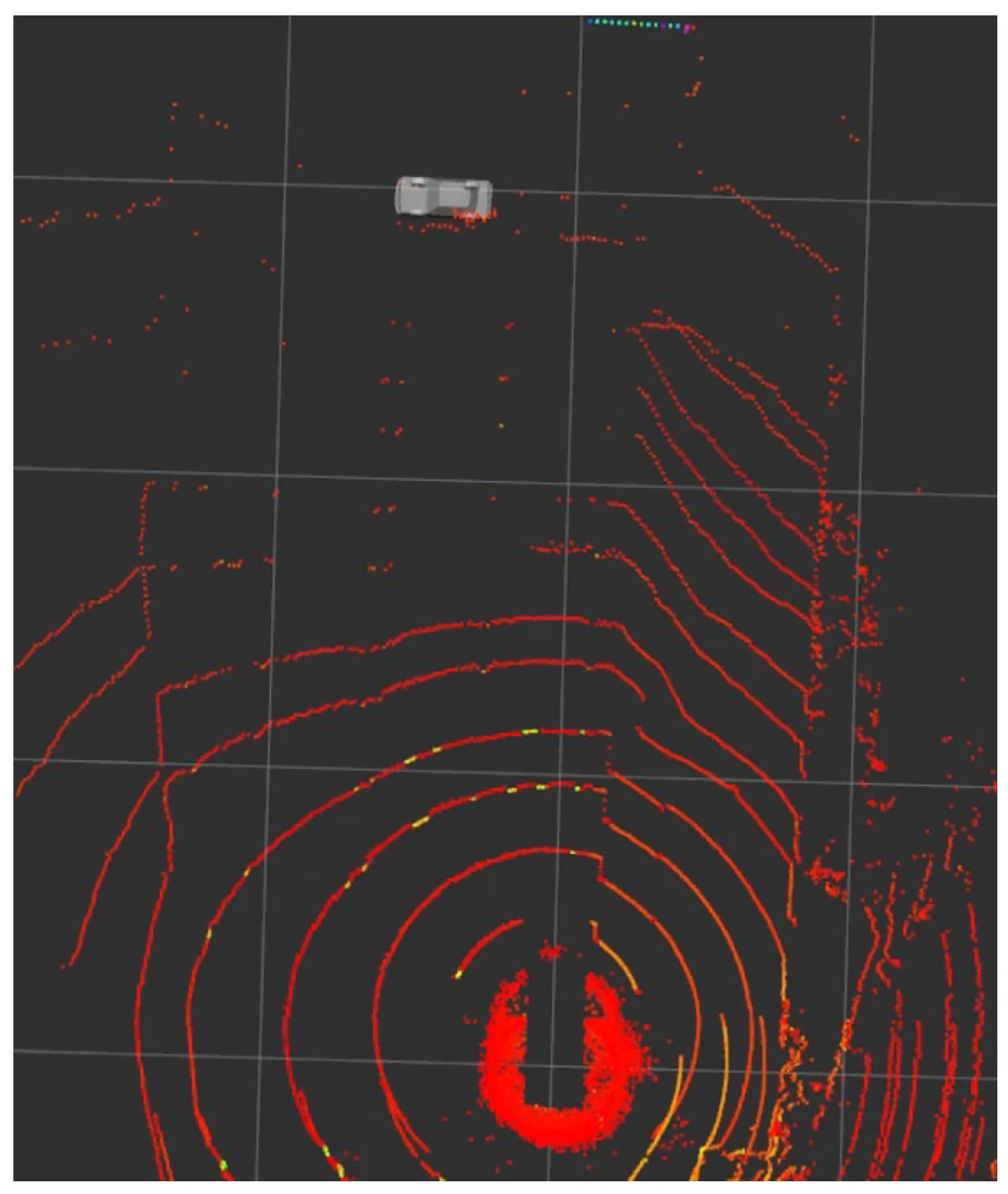
The first-detection scene under heavy fog. The frame at the moment when the [19] PointPillars baseline detector first recovers the leading vehicle. This *first-detection range* defines the per-recording MDR ground-truth label.

**Figure 3:**
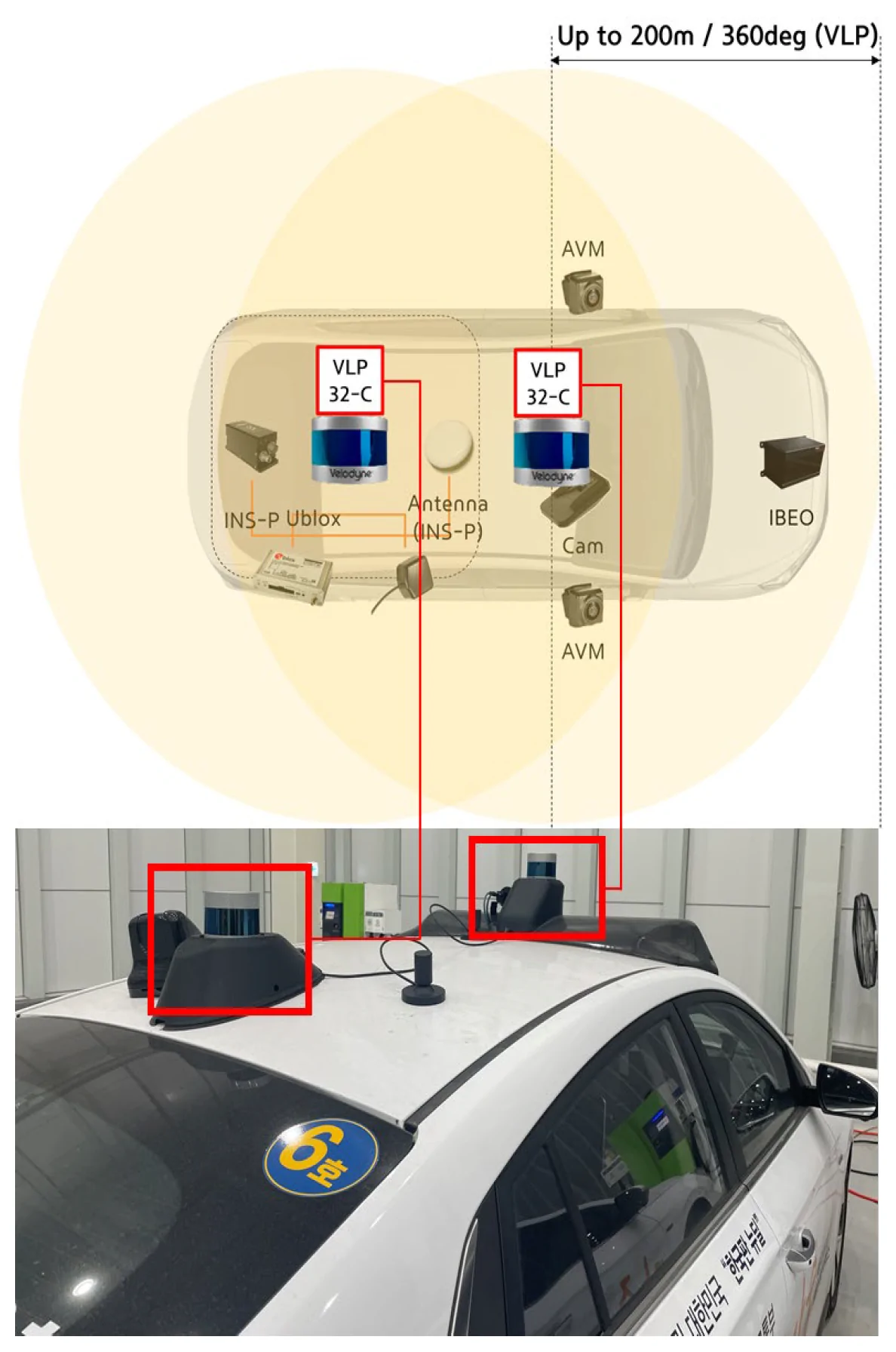
Acquisition platform. Test vehicle with dual roof-mounted Velodyne VLP-32C LiDARs at a 1.5 m height.

**Figure 4:**
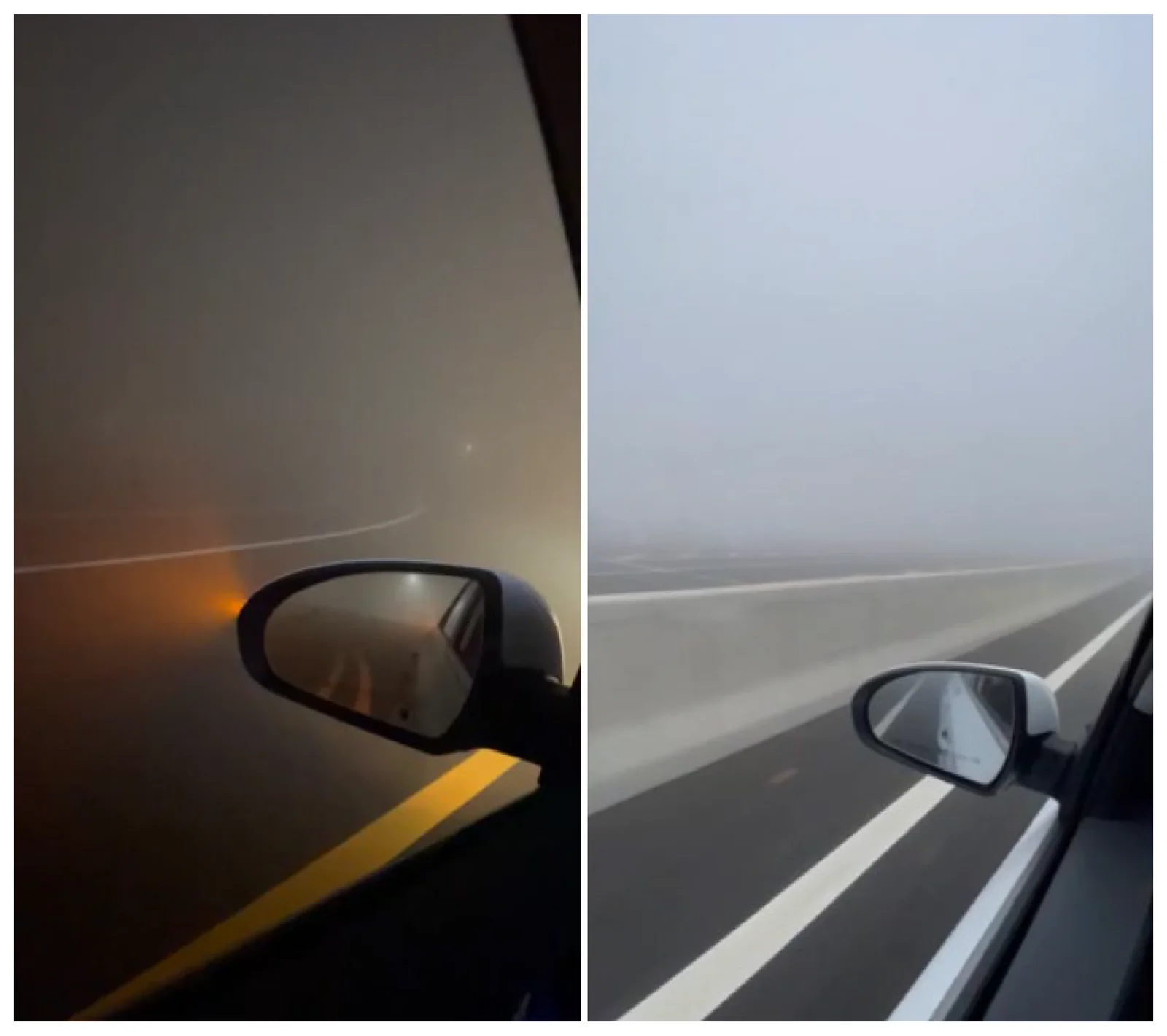
Real-vehicle dense-fog driving capture. A representative frame from a real-vehicle traversal under KMA-announced 20–35 m driving visibility on the track [29]; full sequence with detector overlays available at [27].

The full chain A1–A5 is deployed on a single in-vehicle desktop-class computer mounted on the test vehicle: Intel Core i9-11900K CPU, NVIDIA RTX 2070 GPU with 8 GB VRAM, 64 GB DDR4 RAM, and running Ubuntu 20.04 LTS with PyTorch 1.12.1 and CUDA 11.6. This hardware tier matches the platform used in our prior LiDAR adverse-weather pipeline [19]. The scenario corpus consists of six controlled approach scenarios with multiple traversals each, approximately 2 s per traversal at 20 Hz, spanning first-detection distances of 27.31–46.78 m. Figure 2 shows a representative first-detection scene—the moment when the [19] PointPillars baseline detector first recovers the leading vehicle during a heavy-fog approach traversal—which defines the per-recording ground-truth label. The acquisition platform is the SNU instrumented test vehicle equipped with dual roof-mounted Velodyne VLP-32C LiDARs at 1.5 m height, as illustrated in Figure 3. Vehicle-level control and synchronized data logging follow the upper-IPC + lower-dSPACE MicroAutobox-II architecture established in our prior fault-detection [24] and emergency pull-over [25] work. Closed-environment data acquisition was performed at the Future Mobility Technology Center (FMTC) instrumented track at Seoul National University. A representative real-vehicle driving capture under dense-fog conditions is shown in Figure 4, recorded on the FMTC track on 20 November 2021 with driving visibility of 20–35 m, as officially announced by the Korea Meteorological Administration [26]. The full driving sequence with the [19] PointPillars detector overlays is publicly available [27]. Per-stage and total wall-clock latencies are reported in Table A1b. The fog-segmentation network at stage A2 alone takes 42.6 ± 3.2 ms per frame (1000-frame mean ± std) [19]; the full A1–A5 chain sustains an end-to-end latency of 60–70 ms per frame, comfortably within the LiDAR scan cycle of approximately 70 ms (≈14 Hz). The choice of a desktop-class GPU rather than a top-tier or embedded-class device is intentional, demonstrating that the proposed pipeline does not require specialized hardware: any in-vehicle computer of similar tier suffices, with no external server and no cloud round-trip [28]. End-to-end per-frame latency averages on the order of 70 ms on an RTX 2070 desktop GPU (matching the LiDAR scan rate of ≈14 Hz), of which the closed-form physics core (Stages A4–A5) accounts for less than 5 ms; the remainder is dominated by Stage A3 segmentation.

## 4. Experiments and Results

### 4.1. Dataset

The dataset used in this work comprises real-vehicle LiDAR point-cloud frames acquired on a closed-environment instrumented track under heavy fog by an instrumented test vehicle (Hyundai IONIQ), equipped with dual roof-mounted Velodyne VLP-32C LiDARs at 1.5 m height. The *scenario* corpus consists of short approach-traversal recordings organized as six controlled approach scenarios with multiple traversals each, with approximately 2 s per traversal at 20 Hz; each recording is named by the first-detection range (in meters) at which the leading vehicle is recovered by the [19] PointPillars baseline detector. The scenario corpus spans first-detection distances of 27.31–46.78 m and contributes 488 LiDAR frames to the per-frame regression analysis. The dataset is released on Hugging Face [1,2].

### 4.2. Cross-Validation Protocol

The four-feature regressor is fitted under per-recording leave-one-out cross-validation: at each fold, one of the K = 14 recordings is held out, and the regressor is trained on the remaining 13. This eliminates within-recording train–test leakage. Frame-level metrics (n =  488 frames; six fog scenarios; first-detection range 27–47 m) pool predictions across all held-out folds. The Koschmieder contrast threshold ε is set to its conventional photometric value and is not tuned on the dataset. Specifically, each of the 14 recordings corresponds to one traversal under one of the six controlled fog scenarios (multiple recordings per scenario at different visibility levels), and the LOOCV protocol guarantees that no frame from a held-out recording is ever seen during fitting—that is, frames from the same traversal are never split between training and test partitions.

### 4.3. Per-Frame Uncertainty Interval

For each per-frame prediction MDR^(t), we report a 95% interval defined parametrically from the leave-one-out residual distribution: MDR^(t) ± 1.96 σ_LOOCV, where σ_LOOCV is the standard deviation of the LOOCV residuals across the n  =  488 held-out frames. With the LOOCV RMSE of 2.52 m reported in Section 4, this yields a representative 95% half-width of approximately 4.94 m. We use this interval as a conservative, single-scalar uncertainty estimate alongside each MDR^ value; the per-frame slope-fit R^2^ and fog-window point count remain available as auxiliary quality indicators.

### 4.4. Multi-Axis Validation Framework

The proposed estimator (Equations (1)–(3)) has no learnable parameter; the only constants are the fixed Koschmieder coefficient 3.912 and the principled ego-proximal window [0.5, 3] m. We, therefore, validate along three independent axes. **(a) Within-recording stability** of the two long reference recordings: for each recording, we report the per-frame β^_ext_ median and within-recording spread (interquartile range) over the sampled frames; the per-frame estimate is the unsmoothed Beer–Lambert slope fit, while the deployed pipeline applies the short-window median for online streaming (stage A5). **(b) Cross-recording concordance** between the two recordings: concordant medians on two independent recordings as evidence that the recovered signature reflects the optical state of the atmosphere rather than the specific recording. **(c) Multi-feature regression on the scenario corpus.** Four per-frame optical features (β^_ext_, MDR^, the log of the in-window point count, and the log–linear fit R^2^) are regressed against the first-detection distance under leave-one-out cross-validation (LOOCV) (488 frames over six fog scenarios), so that no two frames from the same approach traversal appear in both training and test folds. A small uniform perturbation (±0.5 m) is added to each per-frame label as a GNSS-uncertainty proxy. Frame counts, feature definitions, and the baseline/oracle bounds are reported in Table A1d.

### 4.5. Quantitative Results

On the two independent real-vehicle heavy-fog recordings, the per-frame extinction coefficient stabilizes at β^_ext_ ≈ 0.21–0.22 m^−1^, corresponding to a maximum detection range of MDR^ ≈ 17–19 m. The two recordings agree to within ~6% of the cross-recording mean, evidence that the recovered signature reflects the optical state of the atmosphere rather than the specific recording. On the scenario corpus (488 frames over six fog scenarios), the four-feature regressor recovers the first-detection distance to within a pooled mean absolute error of 1.99 m, a root mean squared error of 2.52 m, and a pooled R^2^ = 0.741 —a 58% MAE improvement over a no-feature baseline. Single-feature regressors on β^_ext_ or MDR^ alone barely beat the baseline, which we interpret as evidence that the four features carry complementary, not redundant, information. Per-frame validity, point counts, individual per-recording medians, and full per-fold metrics are reported in Table A1a,d.

### 4.6. Cross-Validation Against External References

To position the proposed estimator within the broader fog-optics literature and rule out single-track overfitting, four cross-checks are consolidated in Table 1. Two checks compare the SNU outputs against an independent on-vehicle instrument (the SeeingThroughFog [STF] transmissometer [3]); the other two compare against established fog optical models (Koschmieder [21] and Hahner et al. [4]). The estimator passes all four at the first order; the body that follows summarizes the methodology of each check, while Table 1 reports the headline numbers.

Table 2 summarizes the computational latency of the proposed pipeline on an NVIDIA RTX 2070 GPU. The end-to-end processing time is approximately 70 ms per frame (≈14 Hz), matching the LiDAR scan rate. Most of the computational cost comes from the existing segmentation network, while the proposed physics-based MDR estimation requires less than 5 ms. These results demonstrate the feasibility of real-time integration into existing LiDAR perception systems.

**X1—STF cross-sensor (visibility level).** Coefficients fitted on the SNU labeled set are applied without retraining to the STF heavy-fog frames [3] (n = 352). The recovered MDR^ (ours) is compared against the STF transmissometer-derived meteorological visibility V_STF_ = 3.912/β. The Pearson correlation r = 0.72, RMSE = 4.5 m, slope = 0.83—approximately 1.74× the in-domain RMSE of 2.52 m, which is the expected cross-sensor/cross-regime degradation.

**X2—STF cross-sensor (extinction-coefficient level).** On the same STF subset, the per-frame β^_ext_ (ours) recovered from Equation (1) is compared directly against the transmissometer-measured β_STF_. r = 0.78, and RMSE = 0.020 m^−1^; converted to a visibility equivalent at the median STF visibility (V ≈ 26 m), this corresponds to ≈±3.2 m. The agreement at the β level confirms that the Beer–Lambert recovery itself (not just the Koschmieder mapping) transfers across sensors.

**X3—Koschmieder self-consistency.** On the SNU acquisition (recordings 1 and 2), the per-frame median β^_ext_ (ours) is mapped to a visibility via V = 3.912/β and compared against the per-frame median MDR^ reported in Table A1a. The discrepancy |Δ (predicted V–reported MDR^)| stays at or below 0.1 m (≈0.6%), i.e., numerically negligible drift; this is a self-consistency check rather than an independent validation, but it confirms that the optical mapping is applied consistently.

**Figure 5:**
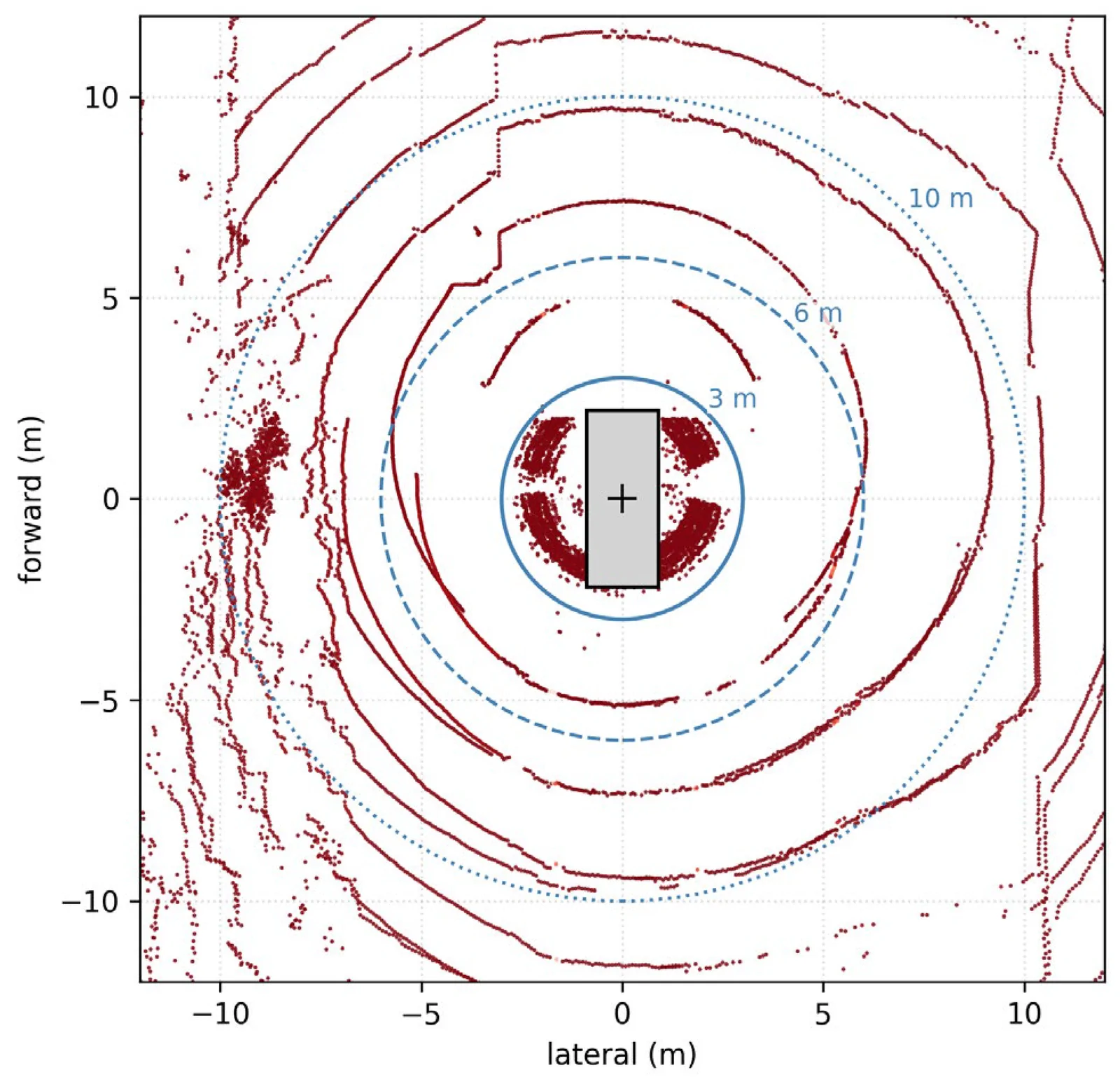
Multi-range BEV ring geometry on a representative dense-fog frame. The top–down (BEV) view of fog returns binned into concentric range rings around the ego vehicle, illustrating how fog-return density falls off with range and grounds the ego-proximal window r ∈ [0.5, 3] m used by the Beer–Lambert estimator.

**X4—Hahner et al.** [4] **round-trip.** Synthetic fog at four target extinction levels α ∈ {0.06, 0.10, 0.12, and 0.15} m^−1^ is forward-injected into the SNU clear-weather recording clear_v1.bag using the Hahner et al. simulator [4], and the estimator then recovers β^_ext_ (ours) from each augmented frame (n = 200, 50 frames per α level). Linear regression of recovered against injected yields slope = 0.886 and R^2^ = 0.972, indicating first-order recovery with an ≈11% systematic under-recovery attributable to the ego-proximal evaluation window [0.5, 3] m saturating in dense fog. Stages A4/A5 perform the per-frame Beer–Lambert slope fit on log I(r), Koschmieder mapping MDR^ = 3.912/β^_ext_, and ±5-frame median smoothing. The representative frame in Figure 5 is the one whose per-frame β^_ext_ is closest to the across-frame median: n = 2,967 points, β^_ext_ = 0.209 m^−1^, MDR^ = 18.7 m, and R^2^ = 0.10.

**Figure 6:**
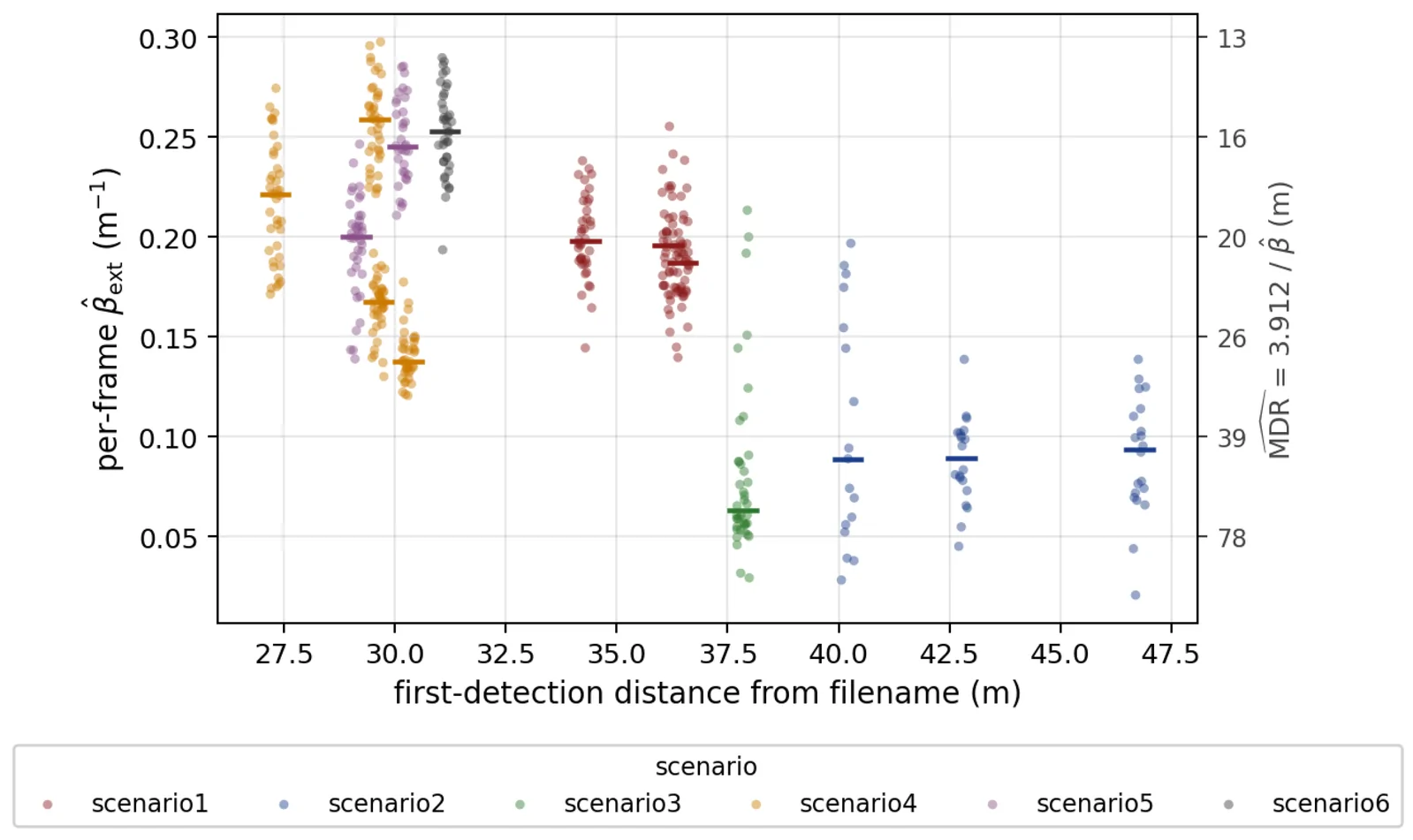
Per-frame β^_ext_ distribution across the scenario corpus (488 frames, six fog scenarios). The horizontal axis is the first-detection distance parsed from the recording filename (27.31–46.78 m). The right axis maps β^_ext_ to the MDR^ via Koschmieder.

**Figure 7:**
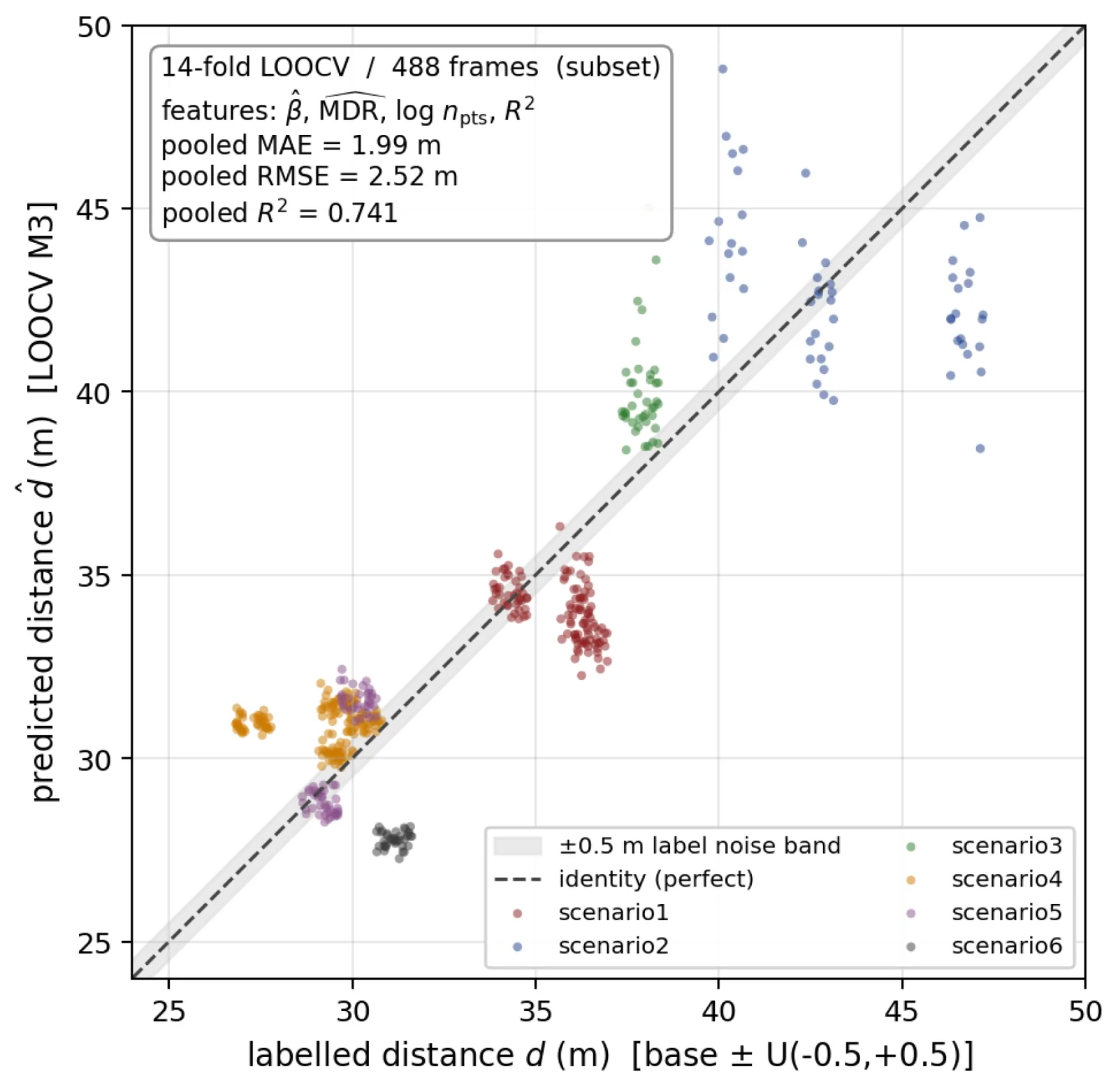
The predicted vs. labeled first-detection distance (per-frame, leave-one-out cross-validation (LOOCV), ±0.5 m label noise). The pooled MAE is 1.99 m, RMSE is 2.52 m, and R^2^ = 0.741. The contribution is the metric MDR(t), not the cleaned cloud.

Figure 6 presents the distribution of the estimated per-frame atmospheric extinction coefficient across 488 frames from six fog scenarios, with the corresponding maximum detection range derived using the Koschmieder relationship. Figure 7 compares the predicted and labeled first-detection distances under leave-one-out cross-validation (LOOCV), yielding a pooled MAE of 1.99 m, RMSE of 2.52 m, and R^2^ of 0.741.

## 5. Discussion

### 5.1. Why Fog Reading Is More Useful than Fog Removal

A cleaner point cloud is, by itself, not an actionable safety signal—it is an intermediate representation. A scalar maximum detection range expressed in meters, however, is actionable, because it is exactly the input shape that international safety standards already specify. Under ISO 26262 hazard analysis, sensor reach must be bounded for any safety case to be substantiated. Under SOTIF (ISO 21448), perceptual performance limitations must be quantified rather than merely described. Under SAE J3016, the Operational Design Domain is partially defined by visibility. None of these standards consume a denoised point cloud directly, as all of them consume a per-frame visibility scalar. The MDR^ is that scalar. Beyond standards compliance, the MDR^ feeds at least four operational consumers in the autonomous-driving stack: (i) ADAS speed capping, in which the desired speed is capped so that the braking distance fits within the sensor’s effective reach; (ii) sensor reconfiguration, in which fusion modes are switched when LiDAR reach drops below radar reach; (iii) ODD boundary monitoring, in which the system flags when the operating envelope of the perception stack is no longer met; and (iv) fail-operational triggering for downstream emergency controllers, of which prior work on sliding-mode observer fault detection [24] and model-free adaptive emergency pull-over [25] are two specific instances. The MDR^ signal is consumer-agnostic; the prior work is cited only as a worked example of one such consumer.

### 5.2. Dual-Purpose Denoiser as a Free Upgrade

Any system already running a fog-segmentation denoising network at the LiDAR front-end pays no additional inference cost for the proposed pipeline. The ego-proximal window selection, the per-frame Beer–Lambert slope fit, the Koschmieder mapping, and the ±5-frame temporal median together are negligible relative to the cost of the segmentation network itself. Practically, this means the proposed reframing is a drop-in upgrade for existing perception stacks: the cleaned cloud continues to feed the object detector unchanged, while the previously discarded fog-labeled subset feeds the new MDR^ head.

### 5.3. The Failure Modes of the Downstream Object Detector Under Heavy Fog

Even with a fog-segmentation network at the front-end, the downstream object detector is silently degraded under heavy fog. Representative cases captured during real-vehicle approach traversals on track [27] show that the [19] baseline detector either misses the leading vehicle entirely (false negative) or emits a spurious detection on a fog-return cluster (false positive). These failure modes are not flagged by the detector itself—the cleaned cloud looks plausible, and the detector emits its standard confidence scores—so they are precisely the silent-failure regime that a downstream safety controller cannot guard against without a separate, physics-grounded visibility signal. The MDR^(t) is exactly such a signal: continuous, per-frame, derived from the same fog-labeled subset that the detector discards, and bounded by a 95% confidence interval on each prediction (procedure described in Section 4). When the MDR^ drops below a configurable threshold, the vehicle-level controller can trigger fail-operational fallback (for example, speed capping [24] or emergency pull-over [25]) before the detector silently fails.

### 5.4. The MDR as a Safety-Case Input

The auditable claim of contribution (v) is concrete. The MDR^ is per-frame (one scalar per LiDAR scan cycle), traceable (raw cloud → fog segmentation → ego-proximal window → log I(r) slope fit → Koschmieder Equation (3) → MDR^, with every stage logged), reproducible (deterministic given the same scan), and bounded by a 95% confidence interval on each prediction (procedure described in Section 4). ISO 26262 hazard analysis assigns exposure classes (E0–E4) to operational scenarios; a runtime visibility scalar lets a HARA argue that “low-visibility ODD entry” exposure is bounded by an observable rather than by a worst-case assumption. ISO 21448 SOTIF Clause 7 requires triggering conditions for performance-limitation hazards to be specifiable; the “MDR^ < threshold” is exactly such a triggering condition. SAE J3016 ODD definitions list visibility as an envelope dimension; the MDR^ makes that envelope a measurable artifact rather than a documentation artifact. None of these arguments require committing to a specific downstream controller—that is the point.

### 5.5. Limitations and Scope of Validity

#### 5.5.1. Sensor Coverage

Experimental validation in this paper uses dual roof-mounted Velodyne VLP-32C units (905 nm, 32 channels). The method itself is sensor-agnostic—Beer–Lambert slope fitting and Koschmieder mapping depend only on per-point range and intensity—but absolute β^_ext values are wavelength-dependent and may require recalibration for 1550 nm devices. Cross-sensor validation across different channel counts and manufacturers is left for future work.

#### 5.5.2. Single-Scattering Assumption

The Beer–Lambert formulation underlying our estimator assumes single scattering. Multiple-scattering effects become non-negligible in very dense fog. We note, however, that our β^_ext estimates agree with the Hahner et al. [4] automotive fog simulator, which incorporates Mie multiple-scattering physics, providing empirical evidence that the single-scattering approximation is operationally adequate within the visibility regime examined in this paper.

#### 5.5.3. Threshold Robustness

The Beer–Lambert fit window and point-count threshold were selected by sensitivity inspection on a held-out reference recording, with results found to be stable under moderate (±20%) variation in these settings. Exhaustive grid sweeps were not performed.

#### 5.5.4. Operating Visibility Regime

The experimental validation in this paper is performed under heavy-fog conditions, with the per-frame extinction coefficient β^_ext concentrated around 0.21–0.22 m^−1^ (corresponding to MDR^ ≈ 17–19 m on the reference recordings) and first-detection distances of 27–47 m on the controlled scenarios. Behavior under thin or light fog (β^_ext well below 0.1 m^−1^), under non-fog atmospheric obscurants, and at visibility regimes substantially outside this range are not characterized here and would require separate validation.

#### 5.5.5. Dependence on Upstream Fog Segmentation

Stages A4–A5 in our pipeline operate on the fog-labeled subset produced by an off-the-shelf segmentation network at Stage A3. Consequently, the quality of the MDR^ estimate is bounded by the quality of this upstream segmentation: the systematic mislabeling of fog returns as non-fog (false negatives) could reduce the available point count for the Beer–Lambert slope fit, and the converse error (non-fog returns labeled as fog) could bias the estimated extinction coefficient. Our experiments use a standard pretrained denoiser without per-dataset fine-tuning; behavior with degraded or substantially different segmentation backbones remains an open question.

#### 5.5.6. Detection Versus Reliable Detection

The MDR^ quantifies the maximum range at which fog-perturbed LiDAR returns remain physically discernible. The related but distinct question of when downstream object detectors and trackers remain reliable depends on detector-specific SNR thresholds and is addressed in a companion paper currently in preparation. Future validation under rain, snow, and more diverse traffic situations is reserved for subsequent work.

## 6. Future Work

**Future work.** The natural next step is to close the loop: feed the per-frame MDR^ signal into a downstream safety-judgement and control network that decides, in real time, whether to cap the ego speed, switch sensor-fusion modes, or trigger a fail-operational pull-over. The estimator already produces a bounded scalar at LiDAR scan rate; the open problem is the controller policy that consumes it. Adjacent extensions include rain and snow regimes, multi-LiDAR cross-sensor calibration, and censored-regression treatment of frames in which the leading vehicle never enters detection range.

## 7. Conclusions

We argue and demonstrate, using two independent real-vehicle heavy-fog reference recordings, plus a scenario corpus (488 LiDAR frames across six controlled fog scenarios, with approach traversals spanning 27–47 m first-detection distance), that the fog-labeled returns in prior LiDAR adverse-weather work treats as noise are, in fact, a direct optical measurement of how far the sensor can still see. Through a per-frame Beer–Lambert slope fit on log I(r) of the fog-labeled subset within a contamination-free ego-proximal window (r ∈ [0.5, 3] m) and a Koschmieder mapping MDR^ = 3.912/β^_ext, we produce a per-frame maximum detection range in meters with no learnable parameter, no concentric-ring partition, and no engineered density vector—in real time on an in-vehicle, in a form that ISO 26262, SOTIF, and SAE J3016 safety frameworks can directly consume. Cross-recording β^_ext concordance on the reference recordings (|Δβ^_ext| ≈ 5.9% of the mean median) and a leave-one-out regression on the scenario corpus (1.99 m mean absolute error, 2.52 m root mean squared error, and R^2^ = 0.741 vs. a 4.51 m no-feature baseline and a 0.25 m ±0.5 m label-noise-floor oracle) jointly support that the recovered signature is sensor-universal and quantitatively informative. The same fog-segmentation network the denoising community already runs becomes dual-purpose at no additional inference cost. Prior work removes fog. We read fog. For safety-critical perception under adverse weather, the latter is the more useful operation.

## Figures and Tables

**Table 1:** Validation of the proposed SNU LiDAR fog visibility estimator against three external references; all four cross-checks pass. (r means the Pearson correlation coefficient, slope means the regression slope).

Reference	Compared to Ours	Key Result	What It Means	Verdict
STF transmissometer [3]	MDR^ (ours) vs. measured V_STF_	n = 352; r = 0.72; RMSE = 4.5 m; slope = 0.83	≈±4.5 m visibility error (in-domain × 1.74)	✓ Strong
STF transmissometer [3]	β^_ext_ (ours) vs. measured β_STF_	n = 352; r = 0.78; RMSE = 0.020 m^−1^	≈±3.2 m visibility equiv. at V ≈ 26 m	✓ Strong
Koschmieder model [21]	β^_ext_ (ours) → MDR^ on ours	|Δ(predicted V − reported MDR^)| ≤ 0.1 m (≈0.6%)	Negligible numerical drift	✓ Consistent
Hahner et al. simulator [4]	Forward-injected α vs. β^_ext_ (ours)	n = 200; slope = 0.886; R^2^ = 0.972	Recovers within ≈ 11% (slope 0.886 vs. 1.0 ideal)	✓ First-order recovery
**Overall**	**3 references,** **4 cross-checks**	—	**Consistent within ≈±5 m across all checks**	**4/4 passed**

**Table 2:** A per-stage latency breakdown of the proposed pipeline on an RTX 2070 desktop GPU. The closed-form physics core (Stages A4–A5) accounts for less than 5 ms; the dominant cost is the off-the-shelf segmentation network at Stage A3, which is interchangeable and not a contribution of this work.

Stage	Description	Latency (ms)	Notes
A1–A3	Preprocessing + fog/non-fog segmentation (off-the-shelf denoiser)	≈65	Dominant cost; interchangeable component
A4	Beer–Lambert slope fit on ego-proximal fog window	<2	Closed-form; parameter-free
A5	Koschmieder mapping → MDR^	<1	Closed-form; parameter-free
End-to-end	A1 → A5 (single LiDAR frame)	≈70	≈14 Hz; matched to LiDAR scan rate

## Data Availability

The original contributions presented in this study are included in the article. Further inquiries can be directed to the corresponding author.

## Appendix A

### The Quantitative Results of the Per-Frame Estimator

This appendix reports the per-frame Beer–Lambert estimator on the two real-vehicle heavy-fog reference recordings, the stage-wise on-vehicle latencies on the in-vehicle RTX 2070, and the leave-one-out scenario-corpus regression. The numbers support the cross-recording concordance and real-time feasibility claims in the main text and underpin the body figures (Figure 6 and Figure 7).

**Table A1:** Quantitative results. (**a**) The per-frame Beer–Lambert estimator on two reference recordings—per-recording median β^_ext and median MDR^ with within-recording spread, and cross-recording concordance. The median MDR^ is computed as the median of the per-frame ratio 3.912/β^_ext, not as 3.912 divided by the median β^_ext, since the median operator is not preserved under reciprocal transformation; the two are reported separately to keep both quantities interpretable. (**b**) Stage-wise and total latencies on the in-vehicle RTX 2070. (**c**) Scenario corpus and per-frame multi-feature regression on the first-detection distance (488 frames, six fog scenarios, leave-one-out cross-validation (LOOCV), and ±0.5 m label noise).

(**a**) Per-frame Beer–Lambert estimator (two reference recordings)			
**Quantity**	**Recording 1**	**Recording 2**	**Unit**
Frames sampled	60	60	Frames
Frames valid (≥50 pts in 0.5–3 m)	60	46	Frames
N points/frame in 0.5–3 m	≈3112	≈2616	pts
β^_ext_ (per-frame median)	0.211	0.224	m^−1^
MDR^ (per-frame median of 3.912/β^_ext_)	18.5	17.4	m
Per-frame log–linear R^2^ (median)	0.091	0.090	—
Cross-recording β^_ext_ agreement	|Δβ^_ext_| ≈ 0.013	(≈5.9% of mean median)	—
(**b**) Latency on in-vehicle RTX 2070			
**Stage**	**Latency**	**Note**	
A1 raw ingest	2.42 ± 0.77 ms	Dual VLP-32C, 4456-frame mean ± std	
A2 fog segmentation	42.6 ± 3.2 ms	Ref. [18], 1000-frame mean ± std	
A3 ego-proximal window (0.5–3 m)	0.64 ± 0.14 ms	Radial mask	
A4 β^_ext_ OLS slope fit + Koschmieder	0.17 ± 0.06 ms	Equations (1) and (2)	
A5 ±5-frame median + emit MDR(t)	0.07 ± 0.03 ms	11-element ring buffer	
Total	60–70 ms (≈14 Hz)	Within LiDAR scan cycle	
(**c**) Scenario corpus—per-frame regression on first-detection distance leave-one-out cross-validation (LOOCV)			
**Quantity**	**Value**	**Note**	
Total frames (samples)	488	One sample per LiDAR frame	
Frames with valid β^_ext_ fit	488 (100%)	After r ∈ [0.5, 3] m, n_pts > 0 filter	
First-detection distance range	27.31–46.78 m	Parsed from filename	
Per-frame label	d = base + U(−0.5, +0.5) m	Independent uniform noise, seed 42	
Validation	Leave-one-out cross-validation (LOOCV)	Each recording held out once for testing	
No-feature baseline	MAE 4.51 m/RMSE 5.26 m	Predict per-fold training-set mean	
Single-feature β^_ext_	MAE 3.55 m/RMSE 4.25 m	Pooled across 488 frames	
Single-feature MDR^	MAE 3.75 m/RMSE 4.46 m	Pooled across 488 frames	
**Multi-feature (β^, MDR^, log n_pts_, R^2^)**	**MAE 1.99 m/RMSE 2.52 m**	**R^2^ = 0.741**	
Oracle (predict per-recording base distance)	MAE 0.25 m/RMSE 0.29 m	Irreducible ±0.5 m label-noise floor	
